# A methodology for apportioning federal SUS resources: the health needs index

**DOI:** 10.11606/s1518-8787.2020054001661

**Published:** 2020-07-28

**Authors:** Áquilas Mendes, Marcel Guedes Leite, Leonardo Carnut

**Affiliations:** I Universidade de São Paulo Faculdade de Saúde Pública Departamento de Política, Gestão e Saúde São PauloSP Brasil Universidade de São Paulo. Faculdade de Saúde Pública. Departamento de Política, Gestão e Saúde. São Paulo, SP, Brasil; II Pontifícia Universidade Católica de São Paulo Programa de Pós-Graduação em Economia Política São PauloSP Brasil Pontifícia Universidade Católica de São Paulo. Programa de Pós-Graduação em Economia Política. São Paulo, SP, Brasil; III Universidade Federal de São Paulo Centro de Desenvolvimento do Ensino Superior em Saúde São PauloSP Brasil Universidade Federal de São Paulo. Centro de Desenvolvimento do Ensino Superior em Saúde. São Paulo, SP, Brasil

**Keywords:** Health Care Rationing, methods. Health Services Needs and Demand, classification. Unified Health System, economy

## Abstract

**OBJECTIVE:**

To present a methodology for apportioning Union resources to the federative units (FU – 26 states and one federal district) within the Brazilian Unified Health System (SUS) based on health needs measured by demographic, socioeconomic, epidemiological and geographical dimensions.

**METHODS:**

The apportionment methodology proposal prioritizes the health needs axis, based on Law 141/2012. We adopted a proxy of needs that measures relative inequalities between, socioeconomic, geographic demographic and epidemiological conditions of the populations of the Brazilian Federative Units (FU) for 2015. We first used an adjustment so that the populations of the 27 FU are corrected by their relative needs regarding age and gender. To calculate the health needs axis, the multivariate techniques factorial analysis and principal components were used, and, based on such correction, we applied the health needs index. Subsequently, this index was applied to simulate the resources that should be transferred by the Ministry of Health to states in 2015.

**RESULTS:**

As we made the methodological choice of transferring a single per capita amount to all states, so the proposal required population correction. Thus, in the analysis of health needs, the FUs that had their population corrected by a factor higher than the national average because of their greater relative need, were the states of: Maranhão, Piauí, Alagoas, Paraíba, Ceará, Pará, Bahia, Acre, Pernambuco, Rio Grande do Norte, Sergipe, Amazonas, Tocantins and Roraima. For the simulation aggregating all the financing blocks, without reducing the resources already distributed to the remaining states in 2015, indicated the additional need of R$ 4.6 billion.

**CONCLUSIONS:**

The proposal addresses the absence of studies presenting quantitative simulations of federal resources distribution within the scope of SUS to the FUs, based on the apportionment criteria defined by Law 141/2012, in order to contribute to the reduction health inequalities and mitigate the effects of the economic crisis.

## INTRODUCTION

The current economic crisis has led to contractions in public spending on the health sector, reducing access to health and negatively impacting it^1^. Recently, Brazil has been facing one of the most intense measures of fiscal austerity, transforming the historic underfunding of the Unified Health System. Health (SUS) in a defunding: The Constitutional Amendment 95/2016 (CA 95). This amendment limited the expansion of public spending over the next 20 years, based on the amount of expenses (2017), corrected by the variation of the broad consumer price index (IPCA). We observe that, from 2017 to 2036, it will generate losses between R$ 162 billion and R$ 400 billion (projection of the gross domestic product of 1.0% and 2.0%, respectively)^4^. In 2018 and 2019, there is already a budget loss of R$ 9.7 billion Reais in the funding of SUS^5^.

Even being subject to budget cuts, health systems are known to have the potential to mitigate some of the negative impacts of an economic crisis. Redistributive financing and reinforced commitments to equity and universal access to health allow health systems to reduce social inequalities and protect the most vulnerable families^2,6^.

Regarding to the allocation of the Ministry of Health (MS) budget in Brazil, since 1990 the Organic Health Law 8080 presented important criteria for the apportionment (article 35) of federal resources to states and municipalities, based on local needs and epidemiological characteristics. However, this article was never regulated during the years of existence of SUS^7^.

Later, Complementary Law 141, of January 13, 2012, made the apportionment gain a prominent position. This law established that the apportionment of Union resources to states and municipalities must be carried out based on the reduction of regional health inequalities^8^. We understand that meeting health needs must be the basis of apportionment, incorporating, in the allocation process, the needs of individuals and different social classes present in a specific territory and guiding public health policy in the sense of universal law^9^.

The apportionment established by this law constitutes a novelty on how resources are historically distributed to state and municipal entities, insofar as it especially considers the criterion of the population’s health needs and also the supply capacity and technical-financial performance of public health actions and services. However the criteria established by Law 141 have not yet been implemented. In addition to difficulties of political and financial nature there is also a lack of more technical and operational studies that present an allocation formula, including the simulation of resources distribution and the determinations of this legal provision.

Thus, we present here the development of a methodology for apportioning Union resources to the federative units (FU – 26 states and one federal district) within the Brazilian Unified Health System (SUS) based on health needs measured by demographic, socioeconomic, geographical and epidemiological dimensions, respecting the criteria established by Law 141/2012. This study seeks to apply a health needs index created to support the apportionment of federal SUS resources. It is important to consider that this methodology as proposed for the FUs should serve as a reference for the apportionment of SUS federal resources to all federal entities (states, Federal District and municipalities). As it is a methodological proposal, investigating the apportionment among the 27 FUs makes it easier to perceive the changes it brings to the distribution of resources as compared to what currently occurs. The methodology of the proposed apportionment criterion shall be the same for the municipalities, i.e. the dimensions that make up the health needs index of the municipalities will be the same, but the indicators that structure them may or may not be different.

## METHODS

Law 141 defines the combination of a set of criteria, which we grouped into three axes, each one corresponding to an allocation index: a) health needs, measured by the demographic, socioeconomic, epidemiological and geographical situation of each federal entity; b) capacity to produce and offer produce health actions and services; c) annual technical and financial performance of health actions and services. In this paper we propose an apportionment methodology for the states, based on the year 2015, which prioritizes the health needs axis.

Then we adopted a proxy of needs that allows measuring relative inequalities between demographic, epidemiological, socioeconomic and geographic conditions of the populations of the different Brazilian states. Before the elaboration and calculation of a health needs index, we proceed to an adjustment so that the populations of the 27 states could be previously corrected by the relative need regarding age and gender, in order to homogenize the differences among states. For this purpose, we used as a proxy for the relative need the corresponding relative use of health services measured by appointments and hospitalizations by age group and gender (each gender and age group presents a differentiated demand for health services). This proposal for the allocation of resources is based on international experiences that use the Population-weighted density concept^10,11^.

Thus, the composition of the final formula for this index can be described by:

- Census population x Population correction factor by age and gender (PCFAG) x population based on the health needs index by socioeconomic, geographic and epidemiological conditions (IHN-SEGE) = Population weighted by Relative Health Needs.

We divided the population into eight age groups by gender (< 1 year, 1–4, 5–14, 15–24, 25–44, 45–54, 55–64 and ≥65). Thus, from the 2015 state populations informed by the Brazilian Institute of Geography and Statistics (IBGE), we sought to define the PCFAG, following the methodology based on the MS study^12^ that defines it as a coefficient standardized by age and gender and used as a proxy for differentiated needs in health services.

As the coefficient of use of health services in primary care and medium and high complexity is very different between genders and age groups, in the case of population correction by age and gender, we propose to take basic appointments (both medical and dental, with psychologists, speech therapists and physiotherapists, extracted from the Ambulatory Information System – AIS/SUS) as marker for care procedures. We justify this providence as the FUs receive federal resources from primary care to serve the municipalities that have not assumed responsibility for this level of care. In the search for a marker for more complex health action procedures, hospital admissions were chosen (extracted from the Hospital Information System – HIS/SUS), as is justified in the work of Mendes et al13. We emphasize that in this work, despite recognizing the bias that the provision of such health services introduces in their use, the only markers effectively available in SUS information systems are appointments and hospitalizations. Thus, for the purpose of this proposal, the resources of primary care are separated from medium and high complexity, as the needs measured by use (markers) are very different among these levels of health care.

For the elaboration of the PCFAG according to age differentials in relation to the frequency of appointments and hospitalizations and by age and gender in Brazil in 2015, we: a) calculated the percentual distribution of the Brazilian population and the FUs according to age and gender, using data from IBGE; b) obtained the 2012 expected average national frequency of appointments and hospitalizations, considering the average national frequencies of appointments and hospitalizations, for each age group and gender, by the respective participation of these strata in the total population of 2015; c) compared the average expected frequency of appointments and hospitalizations per capita in each state with the average Brazilian frequency. The ratio between these two frequencies is the PCFAG, to be used for equitable distribution of resources for health. To correct the population of each FU, the reference population number from IBGE for each state is multiplied by the PCFAG and the population weighted by the age and gender factor is obtained. This should be the population of each FU to be used as a reference for adjustment according to health needs.

The calculation of the health needs axis focused only on socioeconomic, geographic and epidemiological criteria. In order to work with such criteria we used the multivariate techniques principal components and factorial analysis^14^, which were applied in works on equity in the allocation of resources to health^13,15^. Thus, we chose 22 indicators distributed in three dimensions, according to the Chart. We extracted from the IBGE and the Atlas of Human Development Brazil 2013 from the United Nations Development Program (UNPD), the Institute of Applied Economic Research (IPEA) and João Pinheiros Foundation. All data obtained refer to the 2010 population census.

After obtaining the data from the proposed indicators, we calculated Pearson’s 16 linear correlation coefficients among them. The objective of this procedure was to verify the adequacy of the factorial analysis technique to the data as the existence of elevated coefficients is necessary for a positive answer. Still in the preparation of the indicator base for the application of the factor analysis technique, we calculated the measure of sampling adequacy (MSA) for the set of indicators and individually for each one of them^17^.

Once the base indicators were defined, we observed an elevated value of the combined MSA indicators (> 76%), justifying the application of the intended technique^18^. Thus, 12 of the 22 indicators initially proposed remained in the calculation of the health needs index, as shown in the Chart below.

**Chart:** Indicators for the health needs index.

Dimensions/Criteria	N.	Collected indicators	Indicators that composed the index
Epidemiologic	1	Infant mortality rate (less than 1 year)	Infant mortality rate (less than 1 year)
	2	Mortality rate 65 years and older	Mortality rate 65 years and older
	3	General mortality rate	
	4	Fertility rate (population growth)	Fertility rate (population growth)
	5	Life expectancy rate at birth	Life expectancy rate at birth
Socioeconomic	6	% of households with sewage network	% of households with general water network
	7	% of households with general water network	Illiteracy rate
	8	Illiteracy rate	Formal employment rate
	9	Formal employment rate	% population below the poverty line
	10	% population below the poverty line	
	11	% population below the extreme poverty line	Average household income per capita
	12	Average household income per capita	Activity rate of population aged 18 or older
	13	Activity rate of population aged 18 or older	
	14	Gini Index	
	15	Percentage of mothers without elementary school with children up to 15 years old	
	16	Percentage of household with garbage collection	Percentage of people in households with inadequate water supply and sanitation
	17	Percentage of people in households with inadequate water supply and sanitation	% of households with general water network
Geographic	18	Territorial extension	
	19	Demographic density	Demographic
	20	% of rural population	density
	21	Average area per installed municipality	
	22	Municipalities with low population density (less than 22.5 inhabitants per square kilometer - national average)	

For the extraction of the initial factors we considered the principal components analysis (indicated to summarize most of the total variance to a minimum number of factors that could later be used as basic information for the application of other multivariate analysis techniques) and the factorial analysis (indicated to identify latent factors or dimensions that may reflect the common behavior among variables)^19^. Despite frequent doubts about the best way to extract the factors, the literature shows that in most cases the results of the two techniques are similar^17^.

In the aggregation of indicators according to factors, three groupings became evident. A single indicator (demographic density), originally associated with the geographical dimension, started to characterize the third factor (geographical dimension), clearly separating itself from the indicators associated with the second factor. Factor 2, in turn, consists of two indicators: one originally associated with the epidemiological dimension (fertility rate); and the other is associated with the socioeconomic dimension (percentage of people in households with inadequate water supply and sanitation), which does not facilitates its interpretation. Finally, the first factor, the one that brings together the vast majority of indicators, incorporates indicators of the same two dimensions reported in factor 2. Thus, the three factors together comprise more than 92% of the total variance of the indicators involved, each of which is responsible for 44.0%; 33.4% and 22.6% of the total variance, respectively.

Once the factorial structure is known, it is possible to generate from it a score for each unit of the federation, which represents the differentials of health needs according to the socioeconomic, geographic and epidemiological conditions (SEGE) presented by these units. The score is calculated by using the following formula:

$$score_{i}\;=\;0.4395\;\times \;factor\;1\;+\;0.3344\;\times \;factor\;2\;+\;0.2260\;\times \;factor\;3$$

Based on this formula, a score was generated for each unit of the federation, which ranged from -1.2047, for the Federal District, to 1.1047, for the state of Maranhão. However, because it generated both positive and negative scores, it is not possible to establish proportionality among them. To solve this problem, they were normalized to vary between 1 and 2 (2 for Maranhão and 1 for the Federal District), resulting in:

$$\frac{score\;of\;the\;FU\;–\;minimum\;score}{maximum\;score\;–\;minimum\;score}\;+\;1$$

This adjusted score represents the desired proxy for health needs due to socioeconomic, geographical and epidemiological conditions of the FUs, which reflects the relative health needs among them, given by the adopted indicators.

In order to correct the population of the states and the Federal District, already adjusted for demographic conditions, we calculated the average Brazilian score, having as a reference the population adjusted by the PCFAG for each FU. From this average value we created the IHN-SEGE by dividing the adjusted score of each FU by the national average. This is a relative index, assuming the Brazilian mean as one.

Once the health needs index was obtained, we used it as a basis to simulate the resources that should be transferred by the MS to the states, in 2015, based on the financing blocks^20^. The simulation of the apportionment of SUS federal resources was based on the *per capita* form, having as reference the population of each FU estimated by the IBGE (204,450,649 inhabitants), but initially corrected by the population correction index by age and gender (PCIBAG) and then by IHN-SEGE.

## RESULTS

Thus, multiplying the 2015 population estimated by IBGE for the states and the Federal District by PCFAG and IHN-SEGE, we obtain the population weighted by relative health needs, an adjusted number considered as relevant to establish greater equity in the distribution of resources for health according to the population’s different needs (Table 1).

**Table 1:** Estimated population by IBGE. Population Corrected by PCFAG* Correction Factor, Health Needs Index by Socioeconomic, Geographic and Epidemiological Conditions (IHN-SEGE) and Population Corrected by PCFAG and IHN-SEGE, by state, 2015.

Federative Unit	Population - 2015				

	IBGE	Corrected by PCFAG	SCORE_IHN_SEGE_AJUST	INS_SEGE	Corrected by PCFAG and IHN-SEGE
Acre	803,513	762,028	1.7570	1.2471	950,353
Alagoas	3,340,502	3,263,827	1.8674	1.3255	4,326,153
Amapá	766,679	703,302	1.5299	1.0859	763,733
Amazonas	3,938,336	3,687,841	1.6861	1.1968	4,413,581
Bahia	15,203,934	15,029,492	1.6923	1.2012	18,053,617
Ceará	8,905,225	8,853,860	1.7244	1.2240	10,836,984
Distrito Federal	2,914,830	2,867,469	1.0000	0.7098	2,035,359
Espírito Santo	3,929,911	3,883,507	1.2844	0.9117	3,540,407
Goiás	6,610,681	6,367,708	1.3271	0.9420	5,998,195
Maranhão	6,904,241	6,721,912	2.0000	1.4196	9,542,565
Mato Grosso	3,265,486	3,095,397	1.4097	1.0007	3,097,414
Mato Grosso do Sul	2,651,235	2,603,806	1.3604	0.9656	2,514,241
Minas Gerais	20,869,101	21,156,462	1.3371	0.9491	20,079,526
Pará	8,175,113	7,726,893	1.7733	1.2587	9,725,776
Paraíba	3,972,202	4,025,961	1.7657	1.2533	5,045,905
Paraná	11,163,018	11,275,509	1.2537	0.8899	10,034,314
Pernambuco	9,345,603	9,280,738	1.6727	1.1873	11,019,129
Piauí	3,203,262	3,138,979	1.8857	1.3385	4,201,465
Rio de Janeiro	16,550,024	17,260,358	1.2120	0.8603	14,849,491
Rio Grande do Norte	3,442,175	3,413,868	1.6372	1.1621	3,967,169
Rio Grande do Sul	11,247,972	11,873,209	1.2243	0.8690	10,318,411
Rondônia	1,768,204	1,636,106	1.4962	1.0621	1,737,629
Roraima	505,665	461,829	1.6048	1.1391	526,059
Santa Catarina	6,819,190	6,766,324	1.1510	0.8170	5,528,184
São Paulo	44,396,484	44,983,429	1.1633	0.8257	37,144,685
Sergipe	2,242,937	2,162,603	1.6570	1.1762	2,543,596
Tocantins	1,515,126	1,448,236	1.6116	1.1439	1,656,706

Brazil	204,450,649	204,450,649	1.4088	1.0000	204,450,649

Note: PCFAG was calculated from the National Average Number of Primary Care Appointments and the National Average Number of MHC Hospitalizations, by age range and gender.

The states that had their population corrected by a factor higher than the national average, because of having greater relative need, in descending order were: Maranhão, Piauí, Alagoas, Paraíba, Ceará, Pará, Bahia, Acre, Pernambuco, Rio Grande do Norte, Sergipe, Amazonas, Tocantins and Roraima. The states with reduced population in descending order were: Federal District, Santa Catarina, São Paulo, Rio de Janeiro, Paraná, Espírito Santo, Goiás and Rio Grande do Sul (Table 1).

For simulation purposes the resources transferred to the states in 2015, according to the financing blocks, were grouped in two ways. Initially we performed a simulation of the apportionment of total resources by health need, of the primary care, health surveillance and pharmaceutical assistance, blocks. Then we proceed to the same procedure for apportioning these resources, but adding the values transferred to the medium and high complexity (MHC) block to them.

In absolute terms, the resources of the first simulation make up a total of R$ 19.2 billion, which corresponds to approximately 30% of the total federal resources transferred to the FUs by the MS in 2015. Adding the MHC resources, the total reaches R$ 61.9 billion, or 98% of what was transferred. In *per capita* terms, for the first simulation, we found a national average of R$ 94.01 and for the second, R$ 302.98.

Once the population of the states and the Federal District in 2015 was corrected by the proposed health equity apportionment rates (PCFAG and IHN-SEGE), the distribution of MS resources regarding transfers to primary care, pharmaceutical assistance and health surveillance would imply increase to 14 FUs and reduction to the remaining 13, in order to maintain the same total available resources (Table 2). In view of the political difficulties of proceeding to decrease resources for some states and increase for others, we opted to present, in Tables 2, 3 and 4, only the values of the positive additional resources.

**Table 2:** Simulation of the Value of Fund to Fund Transfers for Primary Care, Pharmaceutical Assistance and Health Surveillance, according to PCFAG and IHN-SEGE. 2015.

Federative Unit	Gross value (in R$)			*per capita* (in R$)		Additional resources

	realized	realized pc value x corrected population SEGE	proposed	realized	proposed	
Acre	97,276,355.57	115,053,420.78	111,650,708.54	121.06	138.95	14,374,352.97
Alagoas	403,268,286.25	522,256,920.99	506,811,139.35	120.72	151.72	103,542,853.10
Amapá	76,997,576.69	76,701,732.90	74,433,274.27	100.43	97.09	0.00
Amazonas	383,866,087.99	430,187,827.88	417,464,995.52	97.47	106.00	33,598,907.53
Bahia	1,574,033,439.01	1,869,055,510.20	1,813,778,074.66	103.53	119.30	239,744,635.65
Ceará	981,627,221.98	1,194,565,927.41	1,159,236,564.16	110.23	130.17	177,609,342.18
Distrito Federal	158,546,874.65	110,709,658.50	107,435,413.32	54.39	36.86	0.00
Espírito Santo	335,351,366.46	302,113,794.87	293,178,760.18	85.33	74.60	0.00
Goiás	622,392,887.76	564,727,573.09	548,025,719.21	94.15	82.90	0.00
Maranhão	848,746,742.79	1,173,079,123.70	1,138,385,234.04	122.93	164.88	289,638,491.25
Mato Grosso	328,942,549.64	312,012,150.55	302,784,371.37	100.73	92.72	0.00
Mato Grosso do Sul	284,237,318.36	269,550,303.11	261,578,335.76	107.21	98.66	0.00
Minas Gerais	2,262,993,397.23	2,177,373,854.23	2,112,977,884.06	108.44	101.25	0.00
Pará	762,508,772.93	907,142,163.31	880,313,376.16	93.27	107.68	117,804,603.23
Paraíba	585,155,005.87	743,324,899.10	721,341,017.96	147.31	181.60	136,186,012.09
Paraná	1,009,034,358.76	907,009,857.39	880,184,983.19	90.39	78.85	0.00
Pernambuco	1,011,979,548.45	1,193,195,721.60	1,157,906,882.27	108.28	123.90	145,927,333.82
Piaui	503,015,996.35	659,766,236.99	640,253,608.58	157.03	199.88	137,237,612.23
Rio de Janeiro	1,245,261,349.40	1,117,309,403.79	1,084,264,907.14	75.24	65.51	0.00
Rio Grande do Norte	444,804,577.48	512,645,274.25	497,483,757.67	129.22	144.53	52,679,180.19
Rio Grande do Sul	818,863,896.40	751,190,894.12	728,974,375.67	72.80	64.81	0.00
Rondônia	166,618,167.81	163,737,095.36	158,894,560.36	94.23	89.86	0.00
Roraima	50,910,597.60	52,963,921.25	51,397,509.91	100.68	101.64	486,912.31
Santa Catarina	729,850,999.47	591,675,912.55	574,177,059.82	107.03	84.20	0.00
São Paulo	3,061,154,447.46	2,561,140,131.54	2,485,394,249.32	68.95	55.98	0.00
Sergipe	258,239,610.27	292,855,859.26	284,194,628.61	115.13	126.71	25,955,018.34
Tocantins	215,298,794.65	235,417,318.34	228,454,836.18	142.10	150.78	13,156,041.53

Brasil	19,220,976,227.28	19,806,762,487.07	19,220,976,227.28	94.01	94.01	1,487,941,296.42

Sources: SAGE/MS, IBGE.

To understand the procedures adopted to obtain the proposed values, initially the resources effectively transferred to the FUs were divided by their respective population estimated by IBGE, obtaining values per capita. Then we multiplied each per capita value by the population corrected by the health need index. As it generates a total value that is different from that effectively available we proceeded to a proportional adjustment for each FU to restore the total added value to that observed. Dividing the total resources proposed for each FU by the respective IBGE estimated population for 2015, the proposed per capita amount of transfer is obtained, meeting the criteria of equity for health needs.

Applying the same health equity apportionment procedure to the resources allocated in medium and high complexity, the distribution of MS resources would imply an increase in transfers to 15 states and a reduction to 12 others, in order to maintain the same total available resources (Table 3).

**Table 3:** Simulation of the Value of Fund to Fund Transfers for Medium and High Complexity, according to PCFAG and IHN-SEGE, 2015.

Federative Unit	Gross value (in R$)			*per capita* (in R$)		Additional resources

	realized	Realized pc value vs. Corrected population SEGE	proposed	realized	proposed	
Acre	204,203,660.07	241,521,482.67	243,675,041.44	254.14	303.26	39,471,381.37
Alagoas	765,360,722.21	991,188,615.26	1,000,026,681.80	229.12	299.36	234,665,959.59
Amapá	145,752,075.05	145,192,059.42	146,486,683.94	190.11	191.07	734,608.89
Amazonas	568,004,764.60	636,546,815.55	642,222,670.80	144.22	163.07	74,217,906.20
Bahia	2,876,264,269.31	3,415,364,278.87	3,445,817,833.59	189.18	226.64	569,553,564.28
Ceará	1,874,337,983.01	2,280,927,261.20	2,301,265,455.75	210.48	258.42	426,927,472.74
Distrito Federal	513,523,750.25	358,581,896.68	361,779,240.36	176.18	124.12	0.00
Espírito Santo	780,767,775.23	703,383,791.09	709,655,607.22	198.67	180.58	0.00
Goiás	1,303,821,189.57	1,183,020,870.91	1,193,569,435.54	197.23	180.55	0.00
Maranhão	986,442,018.28	1,363,392,022.67	1,375,548,890.92	142.87	199.23	389,106,872.64
Mato Grosso	648,385,099.11	615,013,258.04	620,497,106.43	198.56	190.02	0.00
Mato Grosso do Sul	695,014,627.77	659,102,065.36	664,979,037.53	262.15	250.82	0.00
Minas Gerais	4,490,095,366.90	4,320,214,220.18	4,358,735,991.04	215.16	208.86	0.00
Pará	1,169,762,695.35	1,391,644,397.67	1,404,053,182.02	143.09	171.75	234,290,486.67
Paraíba	755,651,669.45	959,907,537.86	968,466,682.46	190.23	243.81	212,815,013.01
Paraná	2,612,498,379.77	2,348,345,982.76	2,369,285,325.40	234.03	212.24	0.00
Pernambuco	2,423,743,115.25	2,857,765,178.95	2,883,246,826.34	259.35	308.51	459,503,711.09
Piaui	594,342,030.93	779,551,362.33	786,502,336.85	185.54	245.53	192,160,305.92
Rio de Janeiro	3,637,598,138.74	3,263,831,009.92	3,292,933,397.88	219.79	198.97	0.00
Rio Grande do Norte	686,813,888.57	791,565,357.22	798,623,456.14	199.53	232.01	111,809,567.57
Rio Grande do Sul	2,829,897,171.29	2,596,027,246.67	2,619,175,072.60	251.59	232.86	0.00
Rondônia	333,122,322.99	327,362,149.54	330,281,118.16	188.40	186.79	0.00
Roraima	98,464,493.26	102,435,758.23	103,349,140.44	194.72	204.38	4,884,647.18
Santa Catarina	1,474,026,032.70	1,194,964,038.76	1,205,619,096.26	216.16	176.80	0.00
São Paulo	9,405,440,018.91	7,869,139,012.96	7,939,305,248.82	211.85	178.83	0.00
Sergipe	501,052,096.46	568,216,634.50	573,283,214.51	223.39	255.59	72,231,118.05
Tocantins	348,871,082.52	381,471,223.87	384,872,663.32	230.26	254.02	36,001,580.80

Brasil	42,723,256,437.55	42,345,675,529.15	42,723,256,437.55	208.97	208.97	3,058,374,196.00

Sources: SAGE/MS, IBGE.

Thus, for the purpose of the simulation, when the resources transferred to the medium and high complexity block were added to those of the primary care, pharmaceutical assistance and health surveillance blocks to meet the apportionment criteria, the distribution of MS resources would increase in 14 FUs: Acre, Alagoas, Amazonas, Bahia, Ceará, Maranhão, Pará, Paraíba, Pernambuco, Piauí, Rio Grande do Norte, Roraima, Sergipe and Tocantins (Table 4).

**Table 4:** Simulation of the Value of Fund to Fund Transfers for Primary Care, Pharmaceutical Assistance, Health Surveillance and Medium and High Complexity, according to PCFAG and IHN-SEGE – 2015.

Federative Unit	Gross value (in R$)			*per capita* (in R$)		Additional resources

	realized	Realized pc value vs. Corrected population SEGE	proposed	realized	proposed	
Acre		356,574,903.45	355,380,408.02	375.20	442.28	53,900,392.38
Alagoas	1,168,629,00846	1,513,445,536.25	1,508,375,623.15	349.84	451.54	339,746,614.69
Amapá	222,749,651.74	221,893,792.33	221,150,467.10	290.54	288.45	0.00
Amazonas	951,870,852.59	1,066,734,643.43	1,063,161,173.61	241.69	269.95	111,290,321.02
Bahia	4,450,297,708.32	5,284,419,789.07	5,266,717,434.75	292.71	346.40	816,419,726.43
Ceará	2,855,965,204.99	3,475,493,188.60	3,463,850,583.68	320.71	388.97	607,885,378.69
Distrito Federal	672,070,624.90	469,291,555.18	467,719,468.61	230.57	160.46	0.00
Espírito Santo	1,116,119,141.69	1,005,497,585.97	1,002,129,255.06	284.01	255.00	0.00
Goiás	1,926,214,077.33	1,747,748,444.00	1,741,893,636.19	291.38	263.50	0.00
Maranhão	1,835,188,761.07	2,536,471,146.37	2,527,974,184.98	265.81	366.15	692,785,423.91
Mato Grosso	977,327,648.75	927,025,408.59	923,919,952.76	299.29	282.93	0.00
Mato Grosso do Sul	979,251,946.13	928,652,368.48	925,541,462.47	369.36	349.10	0.00
Minas Gerais	6,753,088,764.13	6,497,588,074.41	6,475,821,710.10	323.59	310.31	0.00
Pará	1,932,271,468.28	2,298,786,560.99	2,291,085,822.62	236.36	280.25	358,814,354.34
Paraíba	1,340,806,675.32	1,703,232,436.97	1,697,526,753.98	337.55	427.35	356,720,078.66
Paraná	3,621,532,738.53	3,255,355,840.14	3,244,450,676.51	324.42	290.64	0.00
Pernambuco	3,435,722,663.70	4,050,960,900.55	4,037,390,528.02	367.63	432.01	601,667,864.32
Piauí	1,097,358,027.28	1,439,317,599.32	1,434,496,008.46	342.58	447.82	337,137,981.18
Rio de Janeiro	4,882,859,488.14	4,381,140,413.71	4,366,463,968.05	295.04	263.83	0.00
Rio Grande do Norte	1,131,618,466.05	1,304,210,631.47	1,299,841,637.41	328.75	377.62	168,223,171.36
Rio Grande do Sul	3,648,761,067.69	3,347,218,140.79	3,336,005,246.31	324.39	296.59	0.00
Rondônia	499,740,490.80	491,099,244.91	489,454,104.44	282.63	276.81	0.00
Roraima	149,375,090.86	155,399,679.48	154,879,103.85	295.40	306.29	5,504,012.99
Santa Catarina	2,203,877,032.17	1,786,639,951.32	1,780,654,860.29	323.19	261.12	0.00
São Paulo	12,466,594,466.37	10,430,279,144.50	10,395,338,601.48	280.80	234.15	0.00
Sergipe	759,291,706.73	861,072,493.77	858,187,974.56	338.53	382.62	98,896,267.83
Tocantins	564,169,877.17	616,888,542.21	614,822,018.36	372.36	405.79	50,652,141.19

Brazil	61,944,232,664.83	62,152,438,016.23	61,944,232,664.83	302.98	302.98	4,599,643,728.99

Sources: SAGE/MS, IBGE.

## DISCUSSION

The distribution of adjusted scores of need for socioeconomic, geographic and epidemiological conditions among the Brazilian FU shows the inequality of healthcare according to the conditions regarding the Brazilian regions, concentrating the highest rates in the North and Northeast regions and the lowest located in the southernmost regions of the country, including the Federal District (Table 1). This situation is not new to the historical situation of social inequality in the country^21^.

According to the simulations carried out based on the IHN-SEGE, and considering the proposition that no federative unit would have its resources reduced, i.e. pursuing equity without decreasing the resources that any FU receives through the current mechanisms regarding primary care, pharmaceutical assistance and health surveillance, additional resources of approximately R$ 1.5 billion would be needed in 2015 (Table 2).

Regarding the simulation of resource distribution applied to medium and high complexity activities, this methodology for allocating the federal resources of SUS would demand additional resources of approximately R$ 3.0 billion. We highlight that such value corresponds to the possibility of additional resources, not reducing the amounts already received by the FUs in 2015 (Table 3). The proposal of not reducing the amounts currently transferred to managers, but allocating additional resources, is supported by the issue raised by Piola^22^, that any proposal to change the apportionment criteria of resources requires political consensus of managers.

Considering the simulation aggregating all the financing blocks, additional resources needed would reach the figure of R$ 4.6 billion in 2015 (Table 4). Such value corresponds to only 4.6% of the total expenditure of the MS in that year^23^, thus requiring a relatively small effort to reallocate resources. This could gradually mitigate the inequalities in the distribution of federal SUS resources, which would mitigate the current scenario of crisis and the system’s defunding caused by CA 95.

This proposal advances in the discussion on the apportionment of resources in the health financing area, based on the study by Porto et al.15 that created subsidies for the structuring of Law 141/2012. Previous work developed for England and Scotland based such study.

One of the oldest proposals for equitable allocation of resources and which remains over time, renewing itself, is that of England^24^, by means of its National Health Service (NHS). By the 1970s, the Resource Allocation Working Party (RAWP) proposed an allocation methodology stating that financial resources should be distributed according to the corrected population based on three factors: differences in the structure of gender and age, differences in the need to use services and regional variations of medical care costs^24^. After being used and criticized in some aspects^15^, in 1985, the English government asked a group of experts to revise the methodology so that the formula would better capture “health needs.”

In the 1990s, Carr-Hill et al.^25^ developed a new methodological proposal centered on the use of health services to estimate the potential demand for services generated by “health needs,” adjusting for the distribution of service offerings. Until now the English experience has served as a reference for any study of equitable methodologies for the apportionment of financial resources in health systems, even with the privatization process that the English NHS has been going through^26^.

The case of Scotland was no different as it has also maintained a determination of equity in the allocation of resources within its health system for several years. However, unlike the English case, this country has been preserving its public health system, not allowing its commercialization, trying to avoid the increase of inequalities associated with greater funding from the private sector^27^.

The Scottish resource allocation formula is used to allocate about 70% of the total Scottish NHS budget, among the 14 territorial councils of that national system^28^. They estimated it as target quotas (percentages) for each NHS council based on a weighted funding approach that begins with the number of people residing in each area of the NHS council. Then the formula adjusts the age and gender profile of each council population, its morbidity and life circumstances (including deprivation) and the excessive costs of providing services in different geographic areas^28^. The most recent formula was developed from 2005 to 2007 and has been adjusted to the present day.

The use of systematic formulas offers the possibility of meeting the equity criteria in the allocation of health resources^28,15,25^, and its improvement occurs over time. In Brazil, the study by Porto et al.^15^ was used as a reference for several investigations on the rate of resources for funding the health area, but almost all of them restricted to investigating specific FUs^29,30^. At the national level, we highlight the article by Nunes^31^, which proposes a self-financing indicator of the FUs as a moderating element, something not contemplated in Law 141/2012 and, therefore, not considered in this proposal.

The accumulation of evidence and the international literature also suggest that contemporary economic crisis and recession may pose a double threat to health and healthcare of populations^1^. First, the increase in poverty, unemployment, homelessness and job insecurity can increase the prevalence of disease as direct effects on the health of the population in the context of a crisis. Second, an economic recession and restrictive policies as crisis responses may cause indirect health effects by reducing access to health care, especially for vulnerable population groups. And it may also widen disparities in access to health care appear in face of diminishing revenues directed to the health system, as well as reductions in the provision of services^1^.

This type of federal resource apportionment, through the application of the health needs index, contributes to the expansion of the debate on the adoption of the new logic of transfers, according to article 17 of Complementary Law 141/2012. Not even did the introduction of MS Ordinance 3,992/201732, which replaced the six financing blocks used for simulations in this study with just two (costing and investment) could change the apportionment criteria. It was only a matter of aggregating the old blocks, in order to make it easier for state and municipal managers or to manage resources transferred by the MS. However groups were created inside to maintain the correspondence with the old funding blocks. Most of these blocks will continue to produce services guided by the historical series of expenditures and financial incentives for the development of specific policies, ratifying the persistence of fragmented logic according to the implementation of health actions and services^33^.

This proposal for a Union resource apportionment methodology, based on the requirements specified by Law 141/2012, presents limitations that must be mentioned. First, we chose to prioritize solely the health needs requirements axis, restricting them to a dimension of equitable resource allocation. We understand that the supply capacity and performance axes demand specific studies, which are still scarce in the Brazilian scope. A second limitation refers to the choice of only classic markers, appointments and hospitalizations – related to the use of basic, medium and high complexity care services, respectively – for population correction by age and gender. Although we must admit that these markers present the supply bias in using such services, they are the only ones effectively available in the SUS information systems.

A third limitation concerns the choice of indicators for calculating the health needs index, since they are restricted to what is made available in information systems. We understand the need to review these indicators periodically, so that they can be improved with regard to better discriminate what constitutes health needs. Another limitation is related to the proposal for apportioning federal resources to the FUs and, as this methodology is adopted by the municipalities, the indicators for calculating the index must be changed to suit each local reality. Finally, it is prudent to remember that the application of the proposed methodology cannot be disconnected from the dynamics of capitalism and its repercussions in the SUS defunding process. Therefore, for really achieving equity in the allocation of resources, it is necessary to have increases in public spending on health, something that is unlikely while CA 95 is in effect.

## CONCLUSIONS

In the scenario of economic crisis and SUS defunding process, the discussion is not restricted to only increasing the resources for this system, but it implies, above all, improving the form of distribution of the Union’s resources to the states and municipalities, improving the apportionment criteria, as established by Law 141/2012. In addition to the proposal discussed in this paper for the states, we recommend that the methodology is extended to the municipalities, along with the appropriate adjustments within the scope of the indicators that integrate the dimensions of the health needs index. Thus, it is not a mere statistical exercise of resources distribution, but a methodological proposal for building the index. Although the magnitude of the budget is an important determinant for the functioning of the health system, decisions about how the available resources are distributed must be equally important to improve equity and universal access to SUS. The adoption of the health needs index advances in the discussion on equity and criteria for apportioning federal resources the other federative units, and this is the only work with an apportionment exercise supported by Law 141/12 to date.
