# Supplementary material for: A methodology for apportioning federal SUS resources: the health needs index
Source: Rev Saude Publica. 2020 Jul 28;54:77. doi: 10.11606/s1518-8787.2020054001661 (PMC7416632; doi:10.11606/s1518-8787.2020054001661)
Supplement: Translation

# Translation: Uma metodologia para rateio dos recursos federais do SUS: o índice de necessidades de saúde

**Authors:** Áquilas Mendes, Marcel Guedes Leite, Leonardo Carnut

**Affiliations:** I Brasil Universidade de São Paulo. Faculdade de Saúde Pública. Departamento de Política, Gestão e Saúde. São Paulo, SP, Brasil; II Brasil Pontifícia Universidade Católica de São Paulo. Programa de Pós-Graduação em Economia Política. São Paulo, SP, Brasil; III Brasil Universidade Federal de São Paulo. Centro de Desenvolvimento do Ensino Superior em Saúde. São Paulo, SP, Brasil

**Keywords:** Alocação de Recursos para a Atenção à Saúde, métodos, Necessidades e Demandas de Serviços de Saúde, Sistema Único de Saúde, economia

## Abstract

**OBJETIVO:**

Apresentar o desenvolvimento de uma metodologia de rateio dos recursos da União para os estados no Sistema Único de Saúde, baseada em necessidades de saúde medidas pelas dimensões demográfica, socioeconômica, epidemiológica e geográfica.

**MÉTODOS:**

A proposta de metodologia de rateio prioriza o eixo necessidades de saúde, baseado na Lei nº 141/2012*.* Adota-se um *proxy* de necessidades que dimensiona desigualdades relativas entre condições demográficas, epidemiológicas, socioeconômicas e geográficas das populações dos estados brasileiros para o ano de 2015. Primeiramente, utiliza-se um ajuste para que as populações dos 27 estados sejam corrigidas pela necessidade relativa referente a idade e sexo. Para o cálculo do eixo necessidades de saúde, recorreu-se às técnicas multivariadas de análise de componentes principais e fatorial e, com base na população ajustada pelo fator de correção populacional por idade e sexo para cada estado, aplicou-se o índice de necessidades de saúde. Posteriormente, aplicamos esse índice para simular recursos que deveriam ser repassados pelo Ministério da Saúde aos estados em 2015.

**RESULTADOS:**

Como metodologicamente decidiu-se pela transferência de um valor *per capita* único para todos os entes federados, a proposta exige a correção populacional. Assim, na análise por necessidades de saúde, os estados que tiveram sua população corrigida por um fator superior à média nacional, por terem necessidade relativa maior, foram: Maranhão, Piauí, Alagoas, Paraíba, Ceará, Pará, Bahia, Acre, Pernambuco, Rio Grande do Norte, Sergipe, Amazonas, Tocantins e Roraima. No caso da simulação agregando todos os blocos de financiamento, sem reduzir os recursos já distribuídos aos demais estados em 2015, seriam necessários R$ 4,6 bilhões de recursos adicionais.

**CONCLUSÕES:**

A proposta preenche a ausência de estudos que apresentem simulações quantitativas de distribuição de recursos federais, no âmbito do Sistema Único de Saúde, para os demais entes federados, baseada nos critérios de rateio definidos pela Lei nº 141/2012, de forma a contribuir na redução das desigualdades de saúde e mitigar os efeitos da crise econômica.

## INTRODUÇÃO

A atual crise econômica vem provocando contrações dos gastos públicos em saúde, reduzindo o acesso à saúde e impactando negativamente a área^1^ Recentemente, o Brasil se depara com uma das mais intensas medidas de austeridade fiscal, transformando o histórico subfinanciamento do Sistema Único de Saúde (SUS) em um desfinanciamento: a Emenda Constitucional nº 95/2016 (EC 95). Essa emenda limitou a expansão dos gastos públicos pelos próximos 20 anos, baseada no valor das despesas (2017), corrigidas pela variação do índice nacional de preços ao consumidor Amplo (IPCA). Constata-se que, de 2017 a 2036, ela gerará perdas entre R$ 162 bilhões e R$ 400 bilhões (projeção do produto interno bruto de 1,0% e 2,0%, respectivamente)^4^. Em 2018 e 2019, já se verifica a perda orçamentária de R$ 9,7 bilhões de reais no financiamento do SUS^5^.

Mesmo sujeitos a cortes, é sabido que os sistemas de saúde têm o potencial de mitigar alguns dos impactos negativos de uma crise econômica. Um financiamento redistributivo e compromissos reforçados com a equidade e o acesso universal à saúde fazem os sistemas de saúde reduzirem as desigualdades sociais e protegerem as famílias mais vulneráveis^2,6^.

No Brasil, no que diz respeito à alocação do orçamento do Ministério da Saúde (MS), a Lei Orgânica da Saúde nº 8080 já apresentava, desde 1990, critérios de rateio importantes (artigo 35) dos recursos federais a estados e municípios, baseados em necessidades locais e características epidemiológicas. Contudo, esse artigo nunca foi regulamentado ao longo dos anos de existência do SUS^7^.

Com a Lei Complementar nº 141, de 13 de janeiro de 2012, o rateio ganha posição de destaque. Essa lei estabeleceu que o rateio de recursos da União para estados e municípios deve ser realizado com base na redução das desigualdades regionais de saúde^8^. Entendemos que o atendimento às necessidades de saúde deve ser a base do rateio, incorporando, no processo de alocação, as necessidades dos indivíduos e das distintas classes sociais presentes num território específico e orientando a política pública de saúde no sentido do direito universal^9^.

O rateio, estabelecido por essa lei, constitui novidade na trajetória histórica das formas de distribuição de recursos para os entes estaduais e municipais, na medida em que considera especialmente o critério de necessidades de saúde da população e ainda a capacidade de oferta e o desempenho técnico-financeiro das ações e serviços públicos de saúde. No entanto, até o momento, os critérios estabelecidos na Lei nº 141 não foram implementados, pois, além das dificuldades de natureza política e financeira, percebe-se a ausência de estudos técnicos mais operacionais que apresentem uma fórmula de alocação, com simulação de distribuição de recursos, e que considerem as determinações desse dispositivo legal.

Assim, este artigo apresenta o desenvolvimento de uma metodologia de rateio dos recursos da União para os estados no SUS, baseada apenas no eixo necessidades de saúde, medidas pelas dimensões demográfica, socioeconômica, epidemiológica e geográfica, respeitando os critérios estabelecidos pela Lei nº 141/2012. Este estudo busca aplicar um índice de necessidades de saúde, criado para apoiar o rateio desses recursos federais do SUS. É importante considerar que esta metodologia proposta para os estados deverá servir como referência para o rateio dos recursos federais do SUS a todos os entes federativos (estados, Distrito Federal e municípios). Como se trata de uma proposta metodológica, investigar o rateio entre os 27 estados torna mais fácil a percepção das modificações que ela traz na distribuição dos recursos em comparação com o que ocorre atualmente. A metodologia do critério de rateio proposta será a mesma para os municípios, isto é, as dimensões que compõem o índice de necessidades de saúde dos municípios serão as mesmas, podendo os indicadores que as estruturam serem diferentes.

## MÉTODOS

A Lei nº 141 define a combinação de um conjunto de critérios, os quais agrupamos em três eixos, sendo que cada eixo deve corresponder a um índice de alocação: a) necessidades de saúde, medidas pela situação demográfica, socioeconômica, epidemiológica e geográfica de cada ente; b) capacidade de oferta e produção de ações e serviços de saúde; c) desempenho técnico e financeiro anual das ações e serviços de saúde. Este trabalho sugere uma proposta de metodologia de rateio para os estados, com base no ano de 2015, que prioriza o eixo necessidades de saúde*.*

Adotou-se, então, um *proxy* de necessidades que permite dimensionar desigualdades relativas entre condições demográficas, epidemiológicas, socioeconômicas e geográficas das populações dos distintos estados brasileiros. Antes da elaboração e do cálculo de um *índice de necessidades de saúde*, efetuou-se um ajuste para que as populações dos 27 estados pudessem ser corrigidas previamente pela necessidade relativa referente a idade e sexo, de forma a homogeneizar as diferenças existentes entre os estados. Para tanto, usou-se como *proxy* da necessidade relativa a utilização relativa de serviço de saúde medida pelas consultas e internações por faixa etária e sexo (cada sexo e faixa etária apresenta uma demanda de serviços de saúde diferenciada). Essa proposta de alocação de recursos baseia-se nas experiências internacionais que trabalham com o conceito de população ponderada^10,11^.

Assim, a composição da fórmula final desse índice pode ser descrita por:

- População Censo x Pop. com base na Necessidade relativa corrigida por idade e sexo (FCPIS) x pop. com base no índice de necessidade de saúde por condições socioeconômicos, geográficos e epidemiológicas (INS-SEGE) = População ponderada por Necessidades Relativas de Saúde.

Optou-se por agregar a população em oito faixas etárias por sexo (< 1 ano, 1–4, 5–14, 15–24, 25–44, 45–54, 55–64 e 65 ou mais). Assim, a partir das populações estaduais informadas pelo Instituto Brasileiro de Geografia e Estatística (IBGE) para 2015, buscou-se definir o FCPIS, seguindo a metodologia baseada no estudo do Ministério da Saúde^12^ que o define como um coeficiente de utilização de serviços de saúde padronizado por idade e sexo e usado como *proxy* de necessidade diferenciada por idade e sexo.

Como o coeficiente de utilização de serviços de saúde da atenção básica e da média e alta complexidade é muito distinto entre os sexos e as faixas etárias, propõe-se, no caso de correção populacional por idade e sexo, tomar como marcador para procedimentos da atenção básica as consultas (tanto médicas como odontológicas, com psicólogos, fonoaudiólogos e fisioterapeutas, extraídas do Sistema de Informações Ambulatoriais – SIA/SUS). Esse procedimento se justifica pois os estados recebem recursos federais da atenção básica exatamente para atender aos municípios que não assumiram a responsabilidade desse nível de atenção. Já na busca de um marcador para procedimentos mais complexos de ações de saúde, optou-se pelas internações hospitalares (extraídas do Sistema de Informações Hospitalares – SIH/SUS), conforme justificado no trabalho de Mendes et al^13^. Neste trabalho ressalta-se que, apesar de reconhecer o viés que a oferta desses serviços de saúde provoca na sua utilização, consultas e internações se constituem nos únicos marcadores efetivamente disponíveis nos sistemas informacionais do SUS. Sendo assim, para efeito dessa proposta, separam-se os recursos da atenção básica da média e alta complexidade, uma vez que a necessidade medida pelo uso (marcadores) é muito distinta entre esses níveis de atenção à saúde.

Para a elaboração do fator de correção populacional por necessidades de saúde, segundo diferenciais de idade em relação à frequência de consultas e internações e por faixa etária e sexo no Brasil em 2015, foram seguidos os seguintes passos: a) calculou-se a distribuição percentual da população brasileira e das unidades da federação (UF), segundo faixa etária e sexo, usando dados do IBGE; b) obteve-se a frequência média nacional esperada de consultas e de internações, ponderando-se a frequência média nacional de consultas e a frequência média nacional de internações, de cada faixa etária e sexo, obtida para 2012, pela respectiva participação de cada um desses estratos na população total de 2015; c) comparou-se a frequência média esperada de consultas e internações *per capita* de cada estado com a frequência média brasileira. Assim, a razão entre essas duas frequências consiste no fator de correção populacional por idade e sexo (FCPIS) para distribuição equitativa de recursos para a saúde. Para que a população do estado seja corrigida, multiplica-se a população referencial do IBGE de cada estado pelo FCPIS e obtém-se a população ponderada pelo fator idade e sexo. Essa deverá ser a população de cada estado a ser usada como referência para ajustamento segundo necessidades de saúde*.*

O cálculo do eixo necessidades de saúde centrou-se apenas nos critérios epidemiológico, socioeconômico e geográfico. Para se trabalhar com esses critérios, recorreu-se às técnicas multivariadas de análise de componentes principais e fatorial^14^, utilizadas por trabalhos sobre equidade na alocação de recursos para a saúde^13,15^. Assim, elegeram-se 22 indicadores distribuídos em três dimensões, conforme o Quadro. Os indicadores foram extraídos do IBGE e do Atlas do Desenvolvimento Humano no Brasil de 2013 do Programa das Nações Unidas para o Desenvolvimento, Instituto de Pesquisa Econômica Aplicada e Fundação João Pinheiro. Todos os dados obtidos referem-se ao censo demográfico de 2010.

**Quadro:** Indicadores para o índice de necessidades de saúde.

Dimensões / Critérios	N.	Indicadores coletados	Indicadores que integraram o índice
Epidemiológico	1	Taxa de mortalidade infantil (menor de 1 ano)	Taxa de mortalidade infantil (menor de 1 ano)
	2	Taxa de mortalidade de 65 anos e mais	Taxa de mortalidade de 65 anos e mais
	3	Taxa de mortalidade Geral	
	4	Taxa de fecundidade (crescimento populacional)	Taxa de fecundidade (crescimento populacional)
	5	Taxa de expectativa de vida ao nascer	Taxa de expectativa de vida ao nascer
Socioeconômico	6	% de domicílios com rede de esgoto	
	7	% de domicílios com rede geral de água	% de domicílios com rede geral de água
	8	Taxa de analfabetismo	Taxa de analfabetismo
	9	Taxa de emprego formal	Taxa de emprego formal
	10	% população abaixo da linha de pobreza	% população abaixo da linha de pobreza
	11	% população abaixo da linha de extrema pobreza	
	12	Renda média domiciliar per capita	Renda média domiciliar per capita
	13	Taxa de atividade de população com 18 ou mais anos	Taxa de atividade de população com 18 ou mais anos
	14	Índice de Gini	
	15	Porcentagem de mães sem ensino fundamental com filhos até 15 anos	
	16	Porcentagem de domicílio com coleta de lixo	
	17	Porcentagem de pessoas em domicílios com abastecimento de água e esgotamento sanitário inadequados	Porcentagem de pessoas em domicílios com abastecimento de água e esgotamento sanitário inadequados
Geográfico	18	Extensão territorial	
	19	Densidade demográfica	Densidade demográfica
	20	% população rural	
	21	Área média por município instalado	
	22	Municípios com baixa densidade demográfica (menor que 22,5 habitantes por quilômetro quadrado - média nacional)	

Após obter os dados dos indicadores propostos, calcularam-se os coeficientes de correlação linear de Pearson^16^ entre eles. O objetivo desse procedimento foi verificar a adequação da técnica da análise fatorial aos dados, dado que, para uma resposta positiva, é necessária a existência de coeficientes elevados. Ainda na preparação da base dos indicadores para a aplicação da técnica de análise fatorial, calculou-se a *measure of sampling adequacy* (MSA) para o conjunto dos indicadores e individualmente para cada um deles^17^.

Uma vez definidos os indicadores-base, observou-se um valor elevado da MSA conjunta dos indicadores (> 76%), justificando a aplicação da técnica pretendida^18^. Dessa forma, permaneceram no cálculo do índice de necessidades em saúde 12 dos 22 indicadores inicialmente propostos, conforme o Quadro a seguir.

Para a extração dos fatores iniciais, foram consideradas a análise dos componentes principais (indicada para resumir a maior parte da variância total a um número mínimo de fatores que possam ser posteriormente usados como informações básicas para aplicação de outras técnicas de análise multivariada) e a análise fatorial (indicada para identificação de fatores ou dimensões latentes que possam refletir o comportamento comum entre as variáveis)^19^. Apesar das dúvidas frequentes de qual a melhor forma de extrair os fatores, a literatura mostra que na maioria dos casos os resultados das duas técnicas são análogos^17^.

Na agregação dos indicadores segundo fatores, ficam evidentes três agrupamentos. Um único indicador (densidade demográfica), originalmente associado à dimensão geográfica, passou a caracterizar o terceiro fator (dimensão geográfica), separando-se claramente dos indicadores associados ao segundo fator. O fator 2, por sua vez, é constituído de dois indicadores: um associado originalmente à dimensão epidemiológica (taxa de fecundidade) e o outro à dimensão socioeconômica (porcentagem de pessoas em domicílios com abastecimento de água e esgotamento sanitário inadequados), o que não facilita sua interpretação. Por fim, o primeiro fator, aquele que reúne a grande maioria dos indicadores, incorpora indicadores das mesmas duas dimensões relatadas no fator 2. Assim, os três fatores em conjunto comportam mais de 92% da variância total dos indicadores envolvidos, sendo cada um deles responsável por 44,0%; 33,4% e 22,6% da variância total, respectivamente.

Uma vez conhecida a estrutura fatorial, é possível gerar a partir dela um *score* para cada unidade da federação, o qual representa os diferenciais de necessidades de saúde segundo as condições socioeconômicas, geográficas e epidemiológicas (SEGE) apresentadas por essas unidades. O cálculo do *score* é efetuado a partir da fórmula:

$$score_{i}\;=\;0,4395\;\times \;fator\;1\;+\;0,3344\;\times \;fator\;2\;+\;0,2260\;\times \;fator\;3$$

Com base nessa fórmula, foi gerado um *score* para cada unidade da federação, o qual variou de -1,2047, para o Distrito Federal, até 1,1047, para o Maranhão. Entretanto, por gerar *scores* positivos e negativos, não é possível estabelecer uma proporcionalidade entre eles. Para contornar esse problema, eles foram normalizados para variar entre 1 e 2 (2 para o Maranhão e 1 para o Distrito Federal), resultando em:

$$\frac{score\;da\;UF\;–\;score\;mínimo}{score\;máximo\;–\;score\;mínimo}\;+\;1$$

Esse score ajustado representa a *proxy* desejada das necessidades de saúde por condições socioeconômicas, geográficas e epidemiológicas das unidades da federação, refletindo as necessidades relativas de saúde entre elas, dada pelos indicadores adotados.

Para corrigir a população dos estados e Distrito Federal, já ajustada pelas condições demográficas, foi calculado o *score* médio brasileiro, tendo como referência a população ajustada pelo fator de correção populacional por idade e sexo (FCPIS) para cada UF. A partir desse valor médio, foi criado o *í*ndice de necessidades de saúde por condição socioeconômica, geográfica e epidemiológica (INS-SEGE) dividindo o *score* ajustado de cada UF pela média nacional – logo, trata-se de um índice relativo, assumindo a média brasileira como um.

Uma vez obtido o índice de necessidades de saúde, ele foi usado como base para uma simulação dos recursos que deveriam ser repassados pelo Ministério da Saúde aos estados, em 2015, com base nos blocos de financiamento^20^. A simulação do rateio dos recursos federais do SUS se baseou na forma *per capita*, tendo como referência a população de cada unidade federada estimada pelo IBGE (204.450.649 habitantes), mas corrigida inicialmente pelo índice de correção populacional por idade e sexo (ICPIS) e em seguida pelo INS-SEGE.

## RESULTADOS

Assim, multiplicando-se a população estimada pelo IBGE para 2015 para os estados e Distrito Federal e corrigida pelo FCPIS e pelo INS-SEGE, temos uma nova população ajustada pelos fatores considerados até aqui como relevantes para estabelecer maior equidade na distribuição de recursos para a saúde segundo as diferentes necessidades da população (Tabela 1).

**Tabela 1:** População Estimada pelo IBGE, População Corrigida pelo Fator de Correção Populacional por Idade e Sexo - FCPIS*, Índice de Necessidade de Saúde por Condições Socioeconômica, Geográfica e Epidemiológica (INS-SEGE) e População Corrigida pelo FCPIS e INS-SEGE, por estado, 2015.

Estados	População - 2015				

	IBGE	Corrigida pelo FCPIS	SCORE_NS_SEGE_AJUST	INS_SEGE	Corrigida pelo FCPIS e INS-SEGE
Acre	803.513	762.028	1,7570	1,2471	950.353
Alagoas	3.340.502	3.263.827	1,8674	1,3255	4.326.153
Amapá	766.679	703.302	1,5299	1,0859	763.733
Amazonas	3.938.336	3.687.841	1,6861	1,1968	4.413.581
Bahia	15.203.934	15.029.492	1,6923	1,2012	18.053.617
Ceará	8.905.225	8.853.860	1,7244	1,2240	10.836.984
Distrito Federal	2.914.830	2.867.469	1,0000	0,7098	2.035.359
Espírito Santo	3.929.911	3.883.507	1,2844	0,9117	3.540.407
Goiás	6.610.681	6.367.708	1,3271	0,9420	5.998.195
Maranhão	6.904.241	6.721.912	2,0000	1,4196	9.542.565
Mato Grosso	3.265.486	3.095.397	1,4097	1,0007	3.097.414
Mato Grosso do Sul	2.651.235	2.603.806	1,3604	0,9656	2.514.241
Minas Gerais	20.869.101	21.156.462	1,3371	0,9491	20.079.526
Pará	8.175.113	7.726.893	1,7733	1,2587	9.725.776
Paraíba	3.972.202	4.025.961	1,7657	1,2533	5.045.905
Paraná	11.163.018	11.275.509	1,2537	0,8899	10.034.314
Pernambuco	9.345.603	9.280.738	1,6727	1,1873	11.019.129
Piauí	3.203.262	3.138.979	1,8857	1,3385	4.201.465
Rio de Janeiro	16.550.024	17.260.358	1,2120	0,8603	14.849.491
Rio Grande do Norte	3.442.175	3.413.868	1,6372	1,1621	3.967.169
Rio Grande do Sul	11.247.972	11.873.209	1,2243	0,8690	10.318.411
Rondônia	1.768.204	1.636.106	1,4962	1,0621	1.737.629
Roraima	505.665	461.829	1,6048	1,1391	526.059
Santa Catarina	6.819.190	6.766.324	1,1510	0,8170	5.528.184
São Paulo	44.396.484	44.983.429	1,1633	0,8257	37.144.685
Sergipe	2.242.937	2.162.603	1,6570	1,1762	2.543.596
Tocantins	1.515.126	1.448.236	1,6116	1,1439	1.656.706

Brasil	204.450.649	204.450.649	1,4088	1,0000	204.450.649

Nota: O FCPIS foi calculado a partir do Número Médio Nacional de Consultas da Atenção Básica e do Número Médio Nacional de Internações Hospitalares MAC, por faixa etária e sexo.

Ao se considerar a população ponderada por necessidades de saúde, os estados que tiveram sua população corrigida por um fator superior à média nacional, por terem necessidade relativa maior, foram, em ordem decrescente de aumento: Maranhão, Piauí, Alagoas, Paraíba, Ceará, Pará, Bahia, Acre, Pernambuco, Rio Grande do Norte, Sergipe, Amazonas, Tocantins e Roraima. Já os estados com população reduzida por ordem decrescente foram: Distrito Federal, Santa Catarina, São Paulo, Rio de Janeiro, Paraná, Espírito Santo, Goiás e Rio Grande do Sul (Tabela 1).

Os recursos transferidos aos estados em 2015, segundo os blocos de financiamento, foram agrupados de duas maneiras, para efeito de simulação. Inicialmente fez-se uma simulação de rateio do total de recursos, por necessidade de saúde, dos blocos da atenção básica, da vigilância em saúde e da assistência farmacêutica e, em seguida, o mesmo procedimento para o rateio desses recursos, mas adicionando a eles os valores transferidos para o bloco da média e alta complexidade (MAC).

Em termos absolutos, os recursos da primeira simulação perfazem um total de R$ 19,2 bilhões, correspondendo a aproximadamente 30% do total de recursos federais repassados aos estados, em 2015, pelo Ministério da Saúde. Agregando os recursos do MAC, o total atinge R$ 61,9 bilhões, ou 98% do total repassado. Em termos *per capita*, para a primeira simulação, encontramos uma média nacional de R$ 94,01 e para a segunda, R$ 302,98.

Uma vez corrigida a população dos estados e do Distrito Federal em 2015 pelos índices de rateio por equidade em saúde propostos (FCPIS e INS-SEGE), a distribuição dos recursos do MS referente às transferências para atenção básica, assistência farmacêutica e vigilância em saúde implicaria aumento para 14 estados e redução para 13 outros, de forma a manter o mesmo total de recursos disponíveis (Tabela 2). Diante das dificuldades políticas de proceder à diminuição de recursos para alguns estados e ao aumento para outros, optamos por apresentar, nas tabelas 2, 3 e 4, apenas os valores dos recursos adicionais positivos.

**Tabela 2:** Simulação do Valor de Transferências Fundo a Fundo para Atenção Básica, Assistência Farmacêutica e Vigilância em Saúde, segundo FCPI e INS-SEGE. 2015.

UF	Valor Bruto (em R$)			*Per capita* (em R$)		Recursos adicionais

	Realizado	Valor pc realizado x Pop Corrigida SEGE	Proposto	Realizado	Proposto	
Acre	97.276.355,57	115.053.420,78	111.650.708,54	121,06	138,95	14.374.352,97
Alagoas	403.268.286,25	522.256.920,99	506.811.139,35	120,72	151,72	103.542.853,10
Amapá	76.997.576,69	76.701.732,90	74.433.274,27	100,43	97,09	0,00
Amazonas	383.866.087,99	430.187.827,88	417.464.995,52	97,47	106,00	33.598.907,53
Bahia	1.574.033.439,01	1.869.055.510,20	1.813.778.074,66	103,53	119,30	239.744.635,65
Ceará	981.627.221,98	1.194.565.927,41	1.159.236.564,16	110,23	130,17	177.609.342,18
Distrito Federal	158.546.874,65	110.709.658,50	107.435.413,32	54,39	36,86	0,00
Espírito Santo	335.351.366,46	302.113.794,87	293.178.760,18	85,33	74,60	0,00
Goiás	622.392.887,76	564.727.573,09	548.025.719,21	94,15	82,90	0,00
Maranhão	848.746.742,79	1.173.079.123,70	1.138.385.234,04	122,93	164,88	289.638.491,25
Mato Grosso	328.942.549,64	312.012.150,55	302.784.371,37	100,73	92,72	0,00
Mato Grosso do Sul	284.237.318,36	269.550.303,11	261.578.335,76	107,21	98,66	0,00
Minas Gerais	2.262.993.397,23	2.177.373.854,23	2.112.977.884,06	108,44	101,25	0,00
Pará	762.508.772,93	907.142.163,31	880.313.376,16	93,27	107,68	117.804.603,23
Paraíba	585.155.005,87	743.324.899,10	721.341.017,96	147,31	181,60	136.186.012,09
Paraná	1.009.034.358,76	907.009.857,39	880.184.983,19	90,39	78,85	0,00
Pernambuco	1.011.979.548,45	1.193.195.721,60	1.157.906.882,27	108,28	123,90	145.927.333,82
Piaui	503.015.996,35	659.766.236,99	640.253.608,58	157,03	199,88	137.237.612,23
Rio de Janeiro	1.245.261.349,40	1.117.309.403,79	1.084.264.907,14	75,24	65,51	0,00
Rio Grande do Norte	444.804.577,48	512.645.274,25	497.483.757,67	129,22	144,53	52.679.180,19
Rio Grande do Sul	818.863.896,40	751.190.894,12	728.974.375,67	72,80	64,81	0,00
Rondônia	166.618.167,81	163.737.095,36	158.894.560,36	94,23	89,86	0,00
Roraima	50.910.597,60	52.963.921,25	51.397.509,91	100,68	101,64	486.912,31
Santa Catarina	729.850.999,47	591.675.912,55	574.177.059,82	107,03	84,20	0,00
São Paulo	3.061.154.447,46	2.561.140.131,54	2.485.394.249,32	68,95	55,98	0,00
Sergipe	258.239.610,27	292.855.859,26	284.194.628,61	115,13	126,71	25.955.018,34
Tocantins	215.298.794,65	235.417.318,34	228.454.836,18	142,10	150,78	13.156.041,53

Brasil	19.220.976.227,28	19.806.762.487,07	19.220.976.227,28	94,01	94,01	1.487.941.296,42

Fontes: SAGE/MS, IBGE.

Para entender os procedimentos adotados para se chegar aos valores propostos, inicialmente dividiram-se os recursos efetivamente repassados aos estados pela sua respectiva população estimada pelo IBGE para 2015, obtendo um valor *per capita*. Em seguida, tomou-se esse valor *per capita*, multiplicando-o pela população corrigida pelo *índice de necessidade em saúde.* Por gerar um valor totalizado diferente do efetivamente disponível, procedeu-se a um ajuste proporcional de cada UF para restaurar o valor agregado total ao observado. Dividindo os recursos totais propostos para cada estado pela população estimada pelo IBGE para 2015, obtém-se o valor *per capita* proposto de repasse, atendendo aos critérios de equidade por necessidade de saúde.

Aplicando o mesmo procedimento de rateio por equidade em saúde aos recursos alocados na média e alta complexidade, a distribuição dos recursos do MS implicaria aumento dos repasses para 15 estados e redução para 12 outros, de forma a manter o mesmo total de recursos disponíveis (Tabela 3).

**Tabela 3:** Simulação do Valor de Transferências Fundo a Fundo para Média e Alta Complexidade, segundo FCPI e INS-SEGE, 2015.

UF	Valor Bruto (em R$)			Per capita (em R$)		Recursos adicionais

	Realizado	Valor pc realizado x Pop Corrigida SEGE	Proposto	Realizado	Proposto	
Acre	204.203.660,07	241.521.482,67	243.675.041,44	254,14	303,26	39.471.381,37
Alagoas	765.360.722,21	991.188.615,26	1.000.026.681,80	229,12	299,36	234.665.959,59
Amapá	145.752.075,05	145.192.059,42	146.486.683,94	190,11	191,07	734.608,89
Amazonas	568.004.764,60	636.546.815,55	642.222.670,80	144,22	163,07	74.217.906,20
Bahia	2.876.264.269,31	3.415.364.278,87	3.445.817.833,59	189,18	226,64	569.553.564,28
Ceará	1.874.337.983,01	2.280.927.261,20	2.301.265.455,75	210,48	258,42	426.927.472,74
Distrito Federal	513.523.750,25	358.581.896,68	361.779.240,36	176,18	124,12	0,00
Espírito Santo	780.767.775,23	703.383.791,09	709.655.607,22	198,67	180,58	0,00
Goiás	1.303.821.189,57	1.183.020.870,91	1.193.569.435,54	197,23	180,55	0,00
Maranhão	986.442.018,28	1.363.392.022,67	1.375.548.890,92	142,87	199,23	389.106.872,64
Mato Grosso	648.385.099,11	615.013.258,04	620.497.106,43	198,56	190,02	0,00
Mato Grosso do Sul	695.014.627,77	659.102.065,36	664.979.037,53	262,15	250,82	0,00
Minas Gerais	4.490.095.366,90	4.320.214.220,18	4.358.735.991,04	215,16	208,86	0,00
Pará	1.169.762.695,35	1.391.644.397,67	1.404.053.182,02	143,09	171,75	234.290.486,67
Paraíba	755.651.669,45	959.907.537,86	968.466.682,46	190,23	243,81	212.815.013,01
Paraná	2.612.498.379,77	2.348.345.982,76	2.369.285.325,40	234,03	212,24	0,00
Pernambuco	2.423.743.115,25	2.857.765.178,95	2.883.246.826,34	259,35	308,51	459.503.711,09
Piaui	594.342.030,93	779.551.362,33	786.502.336,85	185,54	245,53	192.160.305,92
Rio de Janeiro	3.637.598.138,74	3.263.831.009,92	3.292.933.397,88	219,79	198,97	0,00
Rio Grande do Norte	686.813.888,57	791.565.357,22	798.623.456,14	199,53	232,01	111.809.567,57
Rio Grande do Sul	2.829.897.171,29	2.596.027.246,67	2.619.175.072,60	251,59	232,86	0,00
Rondônia	333.122.322,99	327.362.149,54	330.281.118,16	188,40	186,79	0,00
Roraima	98.464.493,26	102.435.758,23	103.349.140,44	194,72	204,38	4.884.647,18
Santa Catarina	1.474.026.032,70	1.194.964.038,76	1.205.619.096,26	216,16	176,80	0,00
São Paulo	9.405.440.018,91	7.869.139.012,96	7.939.305.248,82	211,85	178,83	0,00
Sergipe	501.052.096,46	568.216.634,50	573.283.214,51	223,39	255,59	72.231.118,05
Tocantins	348.871.082,52	381.471.223,87	384.872.663,32	230,26	254,02	36.001.580,80

Brasil	42.723.256.437,55	42.345.675.529,15	42.723.256.437,55	208,97	208,97	3.058.374.196,00

Fontes: SAGE/MS, IBGE.

Assim, para efeito de simulação, ao serem agregados os recursos repassados ao bloco da média e alta complexidade aos dos blocos da atenção básica, assistência farmacêutica e vigilância em saúde para atender aos critérios de rateio, a distribuição dos recursos do MS aumentaria em 14 estados: Acre, Alagoas, Amazonas, Bahia, Ceará, Maranhão, Pará, Paraíba, Pernambuco, Piauí, Rio Grande do Norte, Roraima, Sergipe e Tocantins (Tabela 4).

**Tabela 4:** Simulação do Valor de Transferências Fundo a Fundo para Atenção Básica, Assistência Farmacêutica, Vigilância em Saúde e Média e Alta Complexidade, segundo FCPI e INS-SEGE - 2015.

UF	Valor Bruto (em R$)			*Per capita* (em R$)		Recursos adicionais

	Realizado	Valor pc realizado x Pop Corrigida SEGE	Proposto	Realizado	Proposto	
Acre	301.480.015,64	356.574.903,45	355.380.408,02	375,20	442,28	53.900.392,38
Alagoas	1.168.629.008,46	1.513.445.536,25	1.508.375.623,15	349,84	451,54	339.746.614,69
Amapá	222.749.651,74	221.893.792,33	221.150.467,10	290,54	288,45	0,00
Amazonas	951.870.852,59	1.066.734.643,43	1.063.161.173,61	241,69	269,95	111.290.321,02
Bahia	4.450.297.708,32	5.284.419.789,07	5.266.717.434,75	292,71	346,40	816.419.726,43
Ceará	2.855.965.204,99	3.475.493.188,60	3.463.850.583,68	320,71	388,97	607.885.378,69
Distrito Federal	672.070.624,90	469.291.555,18	467.719.468,61	230,57	160,46	0,00
Espírito Santo	1.116.119.141,69	1.005.497.585,97	1.002.129.255,06	284,01	255,00	0,00
Goiás	1.926.214.077,33	1.747.748.444,00	1.741.893.636,19	291,38	263,50	0,00
Maranhão	1.835.188.761,07	2.536.471.146,37	2.527.974.184,98	265,81	366,15	692.785.423,91
Mato Grosso	977.327.648,75	927.025.408,59	923.919.952,76	299,29	282,93	0,00
Mato Grosso do Sul	979.251.946,13	928.652.368,48	925.541.462,47	369,36	349,10	0,00
Minas Gerais	6.753.088.764,13	6.497.588.074,41	6.475.821.710,10	323,59	310,31	0,00
Pará	1.932.271.468,28	2.298.786.560,99	2.291.085.822,62	236,36	280,25	358.814.354,34
Paraíba	1.340.806.675,32	1.703.232.436,97	1.697.526.753,98	337,55	427,35	356.720.078,66
Paraná	3.621.532.738,53	3.255.355.840,14	3.244.450.676,51	324,42	290,64	0,00
Pernambuco	3.435.722.663,70	4.050.960.900,55	4.037.390.528,02	367,63	432,01	601.667.864,32
Piauí	1.097.358.027,28	1.439.317.599,32	1.434.496.008,46	342,58	447,82	337.137.981,18
Rio de Janeiro	4.882.859.488,14	4.381.140.413,71	4.366.463.968,05	295,04	263,83	0,00
Rio Grande do Norte	1.131.618.466,05	1.304.210.631,47	1.299.841.637,41	328,75	377,62	168.223.171,36
Rio Grande do Sul	3.648.761.067,69	3.347.218.140,79	3.336.005.246,31	324,39	296,59	0,00
Rondônia	499.740.490,80	491.099.244,91	489.454.104,44	282,63	276,81	0,00
Roraima	149.375.090,86	155.399.679,48	154.879.103,85	295,40	306,29	5.504.012,99
Santa Catarina	2.203.877.032,17	1.786.639.951,32	1.780.654.860,29	323,19	261,12	0,00
São Paulo	12.466.594.466,37	10.430.279.144,50	10.395.338.601,48	280,80	234,15	0,00
Sergipe	759.291.706,73	861.072.493,77	858.187.974,56	338,53	382,62	98.896.267,83
Tocantins	564.169.877,17	616.888.542,21	614.822.018,36	372,36	405,79	50.652.141,19

Brasil	61.944.232.664,83	62.152.438.016,23	61.944.232.664,83	302,98	302,98	4.599.643.728,99

Fontes: SAGE/MS, IBGE.

## DISCUSSÃO

A distribuição dos *scores* ajustados de necessidade por condições socioeconômicas, geográficas e epidemiológicas entre os estados brasileiros evidencia a desigualdade de saúde de acordo com essas condições entre as regiões brasileiras, concentrando os maiores índices nas regiões Norte e Nordeste e os menores nas regiões mais ao sul do país, além do Distrito Federal (Tabela 1). Essa situação não constitui novidade para o histórico quadro de desigualdade social no país^21^.

De acordo com as simulações realizadas com base no INS-SEGE, ao se propor que nenhuma unidade federada tenha seus recursos reduzidos, ou seja, que se possa perseguir a equidade sem diminuir os recursos que cada UF recebe pelos mecanismos atuais de repasse à atenção básica, assistência farmacêutica e vigilância em saúde, seriam necessários recursos adicionais de aproximadamente R$ 1,5 bilhões em 2015 (Tabela 2).

Ao se aplicar a simulação para os recursos distribuídos para a média e alta complexidade, essa metodologia de alocação dos recursos federais do SUS adotada teria demandado novos recursos em cerca de R$ 3,0 bilhões. Também deve-se ressaltar que esse valor corresponde à possibilidade de recursos adicionais, não reduzindo os valores efetivamente recebidos pelos estados em 2015 (Tabela 3). A proposição de não reduzir os valores já transferidos atualmente para os gestores e sim de destinar recursos adicionais encontra respaldo na questão levantada por Piola^22^, de que qualquer proposta de alteração dos critérios de rateios de recursos se defronta com o desafio de ter um consenso político entre os gestores.

No caso da simulação agregando todos os blocos de financiamento, os recursos adicionais necessários teriam atingido a cifra de R$ 4,6 bilhões em 2015 (Tabela 4). Sabe-se que esse valor corresponde a apenas 4,6% do total do gasto do MS nesse ano^23^, o que demandaria um esforço relativamente pequeno de realocação dos recursos. Isso poderia mitigar gradualmente as desigualdades na distribuição dos recursos federais do SUS, o que atenuaria o atual cenário de crise e desfinanciamento do sistema com a EC 95.

Esta proposta avança na discussão sobre rateio de recursos na área de financiamento da saúde, a partir do estudo de Porto et al.^15^, que criou subsídios para a estruturação da Lei nº 141/2012. Tal estudo se baseou em trabalhos anteriores desenvolvidos para a Inglaterra e a Escócia.

Uma das propostas mais antigas de alocação equitativa de recursos e que permanece ao longo do tempo, renovando-se, é a da Inglaterra^24^, por meio de seu *National Health Service* (NHS). Nesse país, na década de 1970, foi proposta pelo *Resource Allocation Working Party* (RAWP) uma metodologia de alocação segundo a qual os recursos financeiros devem ser distribuídos em função da população corrigida a partir de três fatores: diferenças na estrutura de sexo e idade, diferenças nas necessidades de uso de serviços e variações regionais no custo de atenção médica^24^. Depois de utilizada e criticada em alguns aspectos^15^, em 1985, o governo inglês solicitou a um grupo de especialistas a revisão da metodologia para que a fórmula captasse melhor as “necessidades de saúde”.

Nos anos de 1990, Carr-Hill et al.^25^ elaboraram uma nova proposta metodológica centrada em dados de uso de serviços de saúde para estimar a demanda potencial dos serviços gerada pelas “necessidades de saúde”, ajustando pela distribuição da oferta de serviços. Enfim, até o presente momento, a experiência inglesa vem servindo de referência para qualquer estudo de metodologias equitativas de rateio dos recursos financeiros nos sistemas de saúde, mesmo com o processo de privatização que o NHS inglês vem sofrendo^26^.

Não foi diferente no caso da Escócia, que também vem mantendo a determinação de equidade na alocação de recursos da saúde de seu sistema ao longo de vários anos. Contudo, diferentemente do caso inglês, esse país vem preservando seu sistema de saúde público, não permitindo a sua mercantilização, buscando, com isso, evitar o aumento das desigualdades associadas a um maior financiamento do setor privado^27^.

A fórmula de alocação escocesa de recursos é usada na alocação de cerca de 70% do orçamento total do NHS escocês, entre os 14 conselhos territoriais desse sistema nacional^28^. A fórmula calcula as quotas-alvo (percentagens) para cada conselho do NHS com base numa abordagem de captação ponderada que começa com o número de pessoas residentes em cada área dos conselhos do NHS. A fórmula então faz ajustes para o perfil de idade e sexo da população do conselho, suas necessidades adicionais baseadas na morbidade e nas circunstâncias da vida (incluindo a privação) e os custos excessivos de prestação de serviços em diferentes áreas geográficas^28^. A fórmula mais recente foi desenvolvida de 2005 a 2007 e vem sendo ajustada até os dias atuais.

O uso de fórmulas sistemáticas oferece a possibilidade de satisfazer os critérios de equidade na alocação de recursos de saúde^28,15,25^, e seu aprimoramento tem se dado ao longo do tempo. No caso do Brasil, o estudo de Porto et al.^15^ foi usado como referência para diversas investigações sobre rateio de recursos para o financiamento na área da saúde, sendo quase todos eles restritos à investigação no âmbito de estados específicos^29,30^. No âmbito nacional, destaca-se o artigo de Nunes^31^, que propõe a incorporação, nos critérios de rateio, de um indicador de capacidade de autofinanciamento dos estados e do DF, como elemento moderador, algo não contemplado na Lei nº 141/2012 e, portanto, não considerado na proposta aqui apresentada.

O acúmulo de evidências e a literatura internacional também sugerem que a crise econômica e a recessão contemporânea constituem dupla ameaça à saúde e aos cuidados de saúde das populações^1^. Em primeiro lugar, o aumento da pobreza, o desemprego, a falta de moradia e insegurança no trabalho podem aumentar a prevalência de doenças – efeitos diretos na saúde da população no cenário de uma crise. Em segundo lugar, a recessão econômica e políticas restritivas como respostas à crise – efeitos indiretos para a saúde – podem reduzir o acesso aos cuidados de saúde, especialmente para grupos populacionais vulneráveis, e aumentar as disparidades no acesso a cuidados de saúde diante das quedas nas receitas públicas direcionadas para o sistema de saúde, bem como reduções na prestação de seus serviços^1^.

Essa forma de rateio dos recursos federais, por meio da aplicação do *índice de necessidades de saúde,* contribui para a ampliação do debate acerca da adoção da nova lógica dos critérios de transferência, conforme estabelecido no artigo 17 da Lei Complementar nº 141/2012. Tampouco a introdução da Portaria do MS nº 3.992/2017^32^, que substituiu os seis blocos de financiamento utilizados para as simulações neste estudo por apenas dois (custeio e investimento), mudou os critérios de rateio; tratou-se apenas de agregar os antigos blocos, com o intuito de facilitar para os gestores estaduais e municipais o manejo dos recursos repassados pelo MS. No seu interior, foram criados os grupos que mantêm a correspondência aos antigos seis blocos de financiamento. Esses blocos continuam baseados, na sua maioria, em produção de serviços guiados pela série histórica de gastos e incentivos financeiros para o desenvolvimento de políticas específicas, ratificando a persistência da lógica fragmentada conforme a implantação de ações e serviços de saúde^33^.

A presente proposta de uma metodologia de rateio dos recursos da União, com base nos critérios estabelecidos pela Lei nº 141/2012, apresenta limitações que devem ser mencionadas. Em primeiro lugar, optou-se por priorizar apenas os critérios baseados no eixo necessidades de saúde, restringindo-se a uma dimensão da alocação equitativa de recursos. Compreendemos que os eixos capacidade de oferta e desempenho exigem estudos específicos, ainda escassos no âmbito brasileiro. Um segundo aspecto de limitação da proposta refere-se à escolha de apenas marcadores mais clássicos, consultas e internações – relacionados à utilização dos serviços da atenção básica e da média e alta complexidade, respectivamente – para a correção populacional por idade e sexo. Ainda que se possa admitir que esses marcadores apresentam o viés da oferta na utilização desses serviços de saúde, eles são os únicos efetivamente disponíveis nos sistemas de informação do SUS.

Uma terceira limitação diz respeito à escolha dos indicadores para o cálculo do *índice de necessidades em saúde*, uma vez que eles se restringem ao que é disponibilizado nos sistemas de informação. Por sua vez, compreendemos a necessidade de revisão desses indicadores periodicamente, a fim de que possam ser aperfeiçoados no que se refere à melhor discriminação do que constituem as necessidades de saúde. Outra limitação apresentada está relacionada a uma proposta de rateio de recursos federais aos estados e, à medida que essa metodologia for adotada pelos municípios, os indicadores para o cálculo do índice devem ser alterados para se adequar à realidade local. Por fim, é prudente relembrar que a aplicação da metodologia proposta não pode estar desarticulada da dinâmica do capitalismo e sua repercussão no processo de desfinanciamento do SUS. Portanto, para o real alcance da equidade na alocação dos recursos, é necessário haver incrementos no gasto público em saúde, algo pouco provável em tempos de vigência da EC 95.

## CONCLUSÕES

No cenário de crise econômica e processo de desfinanciamento do SUS, sabe-se que a discussão não se restringe a somente incrementar os recursos para esse sistema, mas implica, sobretudo, aprimorar a forma de distribuição dos recursos da União para os estados e municípios, aperfeiçoando os critérios de rateio, conforme estabelecidos pela Lei nº 141/2012. Para além da proposta aqui discutida para os estados, considera-se que a metodologia seja estendida aos municípios, com as devidas adequações no âmbito dos indicadores que compõem as dimensões do índice de necessidades de saúde. Dessa forma, não se trata de um mero exercício estatístico de distribuição de recursos, mas sim de uma proposta metodológica de construção do índice. Embora a magnitude do orçamento seja um importante determinante para o funcionamento do sistema de saúde, decisões sobre como os recursos disponíveis são distribuídos devem ser igualmente importantes para melhorar a equidade e o acesso universal ao SUS. A adoção do índice de necessidades de saúde avança na discussão sobre equidade e critérios de rateio de recursos federais aos demais entes federados, e este se constitui no único trabalho com exercício de simulação de rateio apoiado na Lei nº 141/12 até o momento.
